# A pH-responsive and self-healing CEC/PEG-(CHO)_2_ hydrogel for controlled neuropeptide S delivery and endogenous neural repair after spinal cord injury

**DOI:** 10.3389/fnins.2026.1933351

**Published:** 2026-09-16

**Authors:** Bai Xu, Mengdan Cheng, Yan Gao, Yiqing Wu, Ran Xu, Wentao Shi, Peng Zhao, Xiaojie Lu

**Affiliations:** 1 Neuroscience Center, Wuxi School of Medicine, Jiangnan University, Wuxi, Jiangsu, China; 2 Department of Neurosurgery, Jiangnan University Medical Center, Wuxi, Jiangsu, China; 3 Wuxi Neurosurgical Institute, Wuxi, Jiangsu, China; 4 Department of Neurosurgery, Nanjing Brain Hospital, Nanjing, Jiangsu, China; 5 Department of Neurosurgery, Affiliated Hospital of Jiangnan University, Wuxi, Jiangsu, China; 6 Nanjing Gaochun People’s Hospital, Nanjing, Jiangsu, China; 7 School of Food Science and Technology, Jiangnan University, Wuxi, Jiangsu, China

**Keywords:** CEC/PEG-(CHO)_2_ scaffold, endogenous neural stem/progenitor cells, nerve regeneration, neuropeptide S (NPS), spinal cord injury

## Abstract

A pH-responsive CEC/PEG-(CHO)_2_ hydrogel scaffold was engineered for local delivery of neuropeptide S (NPS) to modulate the hostile microenvironment after spinal cord injury (SCI). The scaffold exhibited accelerated degradation under acidic conditions mimicking the injured site, enabling controlled release of NPS. Its porous microstructure (50−150 μm pore size) and compressive elastic modulus (0.081 MPa, slightly softer than that of a native spinal cord) provide a mechanical microenvironment conducive to neuronal survival and differentiation. In a T11 complete transection mouse model, the NPS-loaded scaffold significantly attenuated local inflammation (reduced MMP-9, TNF-α, CD68; increased IL-10), suppressed glial scar formation, recruited endogenous Nestin^+^ neural stem/progenitor cells (NSPCs), and promoted their differentiation into mature MAP2^+^ neurons with enhanced myelination (MBP^+^) and synaptic integration (SYP^+^/MAP2^+^). These effects were mediated through the NPS-NPSR axis and downstream Ca^2 +^/cAMP signaling pathways. This study demonstrates that a rationally designed pH-responsive hydrogel can effectively remodel the SCI microenvironment and activate endogenous neural repair mechanisms, offering a promising strategy for SCI treatment.

## 1 Introduction

Spinal cord injury (SCI) affects approximately 27 million people globally, with 180,000 new cases annually, resulting in permanent neurological deficits due to the limited regenerative capacity of the central nervous system (Bradbury and Burnside, 2019). The pathological cascade following SCI is characterized by a hostile microenvironment marked by acidosis, intense inflammation, oxidative stress, and glial scar formation that collectively impedes endogenous repair processes (Anjum et al., 2020; Xu et al., 2017). Addressing these pathological features requires therapeutic systems capable of responding dynamically to the injury microenvironment for targeted intervention (Fan et al., 2022; Feng et al., 2025; Yang Y. et al., 2025).

After SCI, the lesion site becomes acidic and assumes an irregular morphology. Microenvironment-responsive or *in situ* forming self-healing drug delivery systems have emerged as promising platforms for SCI repair, enabling spatiotemporally controlled release of therapeutic agents (Wang et al., 2026; Xiao et al., 2025; Zhong et al., 2026). The working principle of pH-responsive hydrogels includes simpler systems neutralize excess H^+^ directly to restore physiological pH, and pH-responsive biomaterial scaffolds release therapeutic cargo to counter excitotoxicity and neuroinflammation. The reaction of hydrogels in an acidic microenvironment is driven by pH-responsive chemical bonds, such as the acid-neutralizing amino groups of poly-L-lysine, Schiff base linkages, phenylboronate esters, succinate esters, metal-phenolic coordination complexes, and the acid-labile degradation of ZIF-8 frameworks (Chen S. et al., 2026; Chen S. et al., 2026; Li et al., 2022; Ren et al., 2026; Tian et al., 2025; Xin et al., 2022; Zhang M. et al., 2025). Furthermore, we note that irregularly shaped lesion cavities can be filled with self-healing hydrogels. The notion of “self-healing” refers to the ability of a material to recover its structure and functions after damage, a property that is particularly valuable in biomaterials design for nerve injury repair (Wu et al., 2016). Self-healing hydrogels can be achieved through various chemical bonds, including covalent types like Schiff bases, disulfides, and acylhydrazones, and non-covalent ones such as hydrogen bonds, metal-ligand coordination, and π-π stacking (Du et al., 2023; Luo et al., 2021, 2026; Zhang M. et al., 2025; Zhao et al., 2026). Among these, the chemical bonds that can simultaneously confer both pH responsiveness and self-healing properties include Schiff base linkages, acylhydrazone bonds, and boronate ester bonds. We selected Schiff base linkages for the preparation of hydrogels aimed at SCI repair. The reaction proceeds under mild conditions with rapid kinetics, exhibits good biocompatibility, and undergoes reversible hydrolysis in an acidic environment. Compared with Schiff base linkages, acylhydrazone bonds exhibit a considerably slower self-healing kinetics, reaching an efficiency of 72.5% after 10 h (Guo et al., 2020). Boronate ester bonds, on the other hand, are highly sensitive to sugars/diols, which are abundant *in vivo*, and given that neural tissues are rich in glycosaminoglycans, their degradation may be further accelerated (Seno et al., 2016).

In our study, we developed the CEC/PEG-(CHO)_2_ hydrogel. It is synthesized via Schiff base reaction between N-carboxyethyl chitosan (CEC) and dibenzaldehyde-terminated poly(ethylene glycol) (PEG-(CHO)_2_). Chitosan is non-toxic, biodegradable, biocompatible, and amenable to sterilization, qualities that render it a highly adaptable material with broad utility in biomedical and clinical fields (Fusco et al., 2013; Upadhyaya et al., 2014). However, native chitosan is only soluble in dilute acidic solutions. In contrast, CEC, synthesized via Michael addition reaction in aqueous solution, exhibits good water solubility and can be used directly at neutral pH, thereby avoiding the use of organic solvents and the associated issue of residual toxicity, while retaining favorable biodegradability and biocompatibility (Ibrahim et al., 2020; Yang S. et al., 2016; Zhou et al., 2013). PEG-(CHO)_2_, a derivative of polyethylene glycol, possesses excellent hydrophilicity and biocompatibility and has been widely employed in biomedical applications. Moreover, the benzaldehyde groups (-CHO) at both termini of the PEG-(CHO)_2_ molecule can undergo Schiff base reactions with the amino groups (-NH_2_) of CEC to form a dynamic crosslinked network (Karakyriazis et al., 2026; Li et al., 2023). The PEG-(CHO)_2_ component not only serves as a crosslinker but also contains arginine-glycine-aspartic acid (RGD)-like sequences that promote cell adhesion, migration, and retention, all critical for recruiting endogenous neural stem/progenitor cells (NSPCs) to the injury site (Qu et al., 2017; You et al., 2018). Critically, the dynamic Schiff base linkages give CEC/PEG-(CHO)_2_ hydrogel pH-responsive degradation and self-healing ability. Under acidic conditions, like those found in the SCI microenvironment, the hydrogel degrades much faster while remaining stable at physiological pH (Wang et al., 2024). This responsiveness allows targeted release of therapeutic drugs or bioactive molecules specifically within the acidic lesion core. Meanwhile, the self-healing hydrogel could serve as an injectable, self-adaptive, and localized drug delivery platform for SCI repair. So far, CEC/PEG-(CHO)_2_ hydrogels have mainly been studied in non-neural applications, such as hepatocellular carcinoma therapy and osteochondral defect repair, where their pH-responsive and self-healing properties are well established (Qu et al., 2017; Wang et al., 2024; You et al., 2018). However, their application in central nervous system repair, especially SCI, remains largely unexplored. Our group recently showed that this platform can work in SCI, delivering exosomes from hypoxia-preconditioned NSPCs to promote blood-spinal cord barrier repair and functional recovery (Gao et al., 2025). Together, these findings suggest that the CEC/PEG-(CHO)_2_ hydrogel is a promising and adaptable platform for SCI repair, worth testing with other bioactive effectors.

The selection of an appropriate bioactive molecule matters just as much as the delivery system. Neuropeptides have drawn growing interest as multifunctional regulators of neural repair (Yang Y. et al., 2025). The concept of neuropeptides, introduced by De Wied in 1974, refers to endogenous peptides synthesized in neurons that regulate intercellular communication and influence a wide range of physiological functions (Eiden et al., 2020). Clinically, neuropeptides such as cholecystokinin and vasopressin are implicated in conditions like epilepsy, where they can exert seizure suppressive effects (Asim et al., 2024; Burtscher and Schwarzer, 2017; Janković and Ðešević, 2022; Kovac and Walker, 2013; Wen et al., 2024). Beyond this, several neuropeptides-including neuropeptide Y, pituitary adenylate cyclase-activating polypeptide (PACAP), urocortin, and galanin-have been shown to promote the survival, proliferation, and neuronal differentiation of NSPCs in various disease models (Cordero-Llana et al., 2014; Matsumoto et al., 2016; Mirchandani-Duque et al., 2022; Rockenstein et al., 2015; Rodrigo et al., 2010). This positions neuropeptides and their receptors as promising targets for therapeutic intervention in neurological disorders. Among these, neuropeptide S (NPS) is particularly noteworthy as it exhibits a broader therapeutic profile than that of many other neuropeptides. Evolutionarily, NPS and its receptor NPSR1 first appear in lungfish and all terrestrial vertebrates, suggesting an adaptive role in land life that may reflect fundamental neural regulatory functions (Pitti and Manoj, 2012; Reinscheid, 2007). This 20 amino acid peptide activates both Gq and Gs signaling pathways, leading to intracellular calcium mobilization and cAMP accumulation, processes known to be important for neural plasticity and survival (Reinscheid and Ruzza, 2021; Shirsath et al., 2024). Beyond its well-characterized roles in arousal and anxiety regulation (Koob and Greenwell, 2004), recent studies have revealed remarkable neuroprotective properties. For instance, NPS enhances synaptic plasticity and reduces Aβ pathology in Alzheimer’s disease models (Zhao et al., 2019); it also alleviates neuropathic pain caused by nerve injury by inhibiting the activation of microglia and astrocytes in the spinal dorsal horn (Yang F. et al., 2016). In Parkinson’s disease, it reduces oxidative stress and protects dopaminergic neurons (Shirsath et al., 2024). Given these multi-faceted properties, we considered NPS a promising candidate for SCI repair. Here, we report its first application in a mouse model of complete spinal cord transection. This distinguishes our work from studies on other neuropeptides (e.g., neuropeptide Y or PACAP), whose neurogenic or neuroprotective effects have been demonstrated predominantly in stroke or Alzheimer’s disease models, without direct validation in SCI.

In this study, we engineered a pH-responsive CEC/PEG-(CHO)_2_ hydrogel scaffold loaded with NPS and characterized its structure and function relationships. In a mouse T11 complete transection SCI model, we tested whether the system could modulate the inflammatory microenvironment, recruit endogenous NSPCs and promote their neuronal differentiation, and ultimately help restore disrupted neural circuits and hindlimb motor function. Together, these findings suggest that the NPS functionalized hydrogel offers a microenvironment responsive strategy for SCI repair.

## 2 Materials and methods

### 2.1 Preparation and characterization of CEC/PEG-(CHO)_2_ hydrogel

To synthesize CEC, chitosan (4.0 g) was dissolved in acrylic acid (5.84 mL) with stirring at 50 °C for 3 days. The mixture was adjusted to pH 10–12 using 1 M NaOH to convert the acrylic acid carboxyl groups into their sodium salts, dialyzed against distilled water for 3 days, and then freeze dried to obtain the CEC polymer. The CEC polymer was dissolved in ddH_2_O to prepare a 4% (wt/vol) stock solution. A 20% (wt/vol) PEG-(CHO)_2_ solution was also prepared. To achieve a molar ratio of -NH_2_/-CHO (*R* = 0.44), the CEC solution (400 μL) and PEG-(CHO)_2_ solution (100 μL) were briefly vortexed at 2800 rpm for 10 s until homogeneity. The mixture was then incubated at 37 °C without agitation until complete gelation occurred.

Hydrogel samples were frozen at −80 °C for 1 h, lyophilized overnight, fractured in liquid nitrogen, and mounted on stubs. Samples were sputter-coated with gold for about 45 s using a Quorum SC7620 instrument, then imaged under a scanning electron microscope.

The swelling properties of CEC/PEG-(CHO)_2_ hydrogel was evaluated. Briefly, samples (*n* = 5 per group) were freeze dried to a constant dry weight (Wd). The dried hydrogels were then immersed in PBS (pH 7.4) at 37 °C. At predetermined time intervals, the samples were removed, their surfaces gently blotted dry, and their swollen weight (Ws) immediately recorded. The swelling ratio (SR) was calculated as SR (%) = (Ws - Wd)/Wd × 100%.

For degradation analysis, hydrogel blocks were placed in 2 mL centrifuge tubes, rinsed three times with PBS (10 mL per rinse), and then incubated in PBS at 37 °C. At designated time points, samples were removed, washed with ddH_2_O, oven dried at 60 °C for 2 days, and weighed to assess mass loss.

Mechanical properties were evaluated by uniaxial compression tests using a mechanical tester. Hydrogel samples (*n* = 5) were compressed at a rate of 1 mm/min until failure. The compressive elastic modulus was calculated from the linear region of the stress-strain curve.

The CEC/PEG-(CHO)_2_ hydrogel was characterized using Fourier-transform infrared spectroscopy (FTIR; Thermo Fisher Nicolet iS20) after freeze-drying to confirm the successful formation of Schiff base bonds.

### 2.2 *In vitro* NPS release study

NPS (1 mg) was mixed with 80 μL of 4% (wt/vol) CEC solution in a 2 mL centrifuge tube. After adding 20 μL of 20% (wt/vol) PEG-(CHO)_2_, the mixture was briefly vortexed and incubated at 37 °C without agitation until gelation was complete. The resulting NPS loaded hydrogel was immersed in 1.5 mL of PBS (PH 7.4) at 37 °C with shaking at 100 rpm. At each predetermined time point, 1 mL of supernatant was collected and replaced with an equal volume of PBS to keep the total volume constant. The collected samples were stored at −20 °C until analysis. NPS concentration in the supernatant was determined using a mouse NPS ELISA kit (Shanghai Youkewei Biotechnology Co., Ltd.). Three independent samples were measured per time point (*n* = 3 per group).

### 2.3 Mouse NSPCs culture

All experimental procedures were approved by the Institutional Animal Care and Use committee of Jiangnan University (Wuxi, China; approval No. JN. No20220615S0721110[309]. NSPCs were freshly isolated from telencephalons of C57BL/6 mouse embryos at embryonic day 15 (E15). Dissected telencephalons were transferred into ice cold PBS. Tissue pieces were then digested with TrypLE (Gibco, USA) for 25 min at 37 °C, followed by the addition of DMEM/F12 medium (Gibco, USA) to stop the enzymatic reaction. The cell suspension was sequentially filtered through 70 μm and 40 μm strainers to remove undigested fragments, followed by centrifugation at 500 *g* for 5 min. The pellet was resuspended in 8 mL of proliferation medium and plated in a T25 flask (Corning, USA). The proliferation medium consisted of DMEM/F12 supplemented with 1% non-essential amino acids, 1% sodium pyruvate, 1% penicillin-streptomycin, 2% B27, 6 g/L glucose, 20 ng/mL epidermal growth factor, 20 ng/mL basic fibroblast growth factor, and 1.83 μg/mL heparin. After about 7 days, neurospheres had formed, and the cultures were used for subsequent experiments.

### 2.4 *In vitro* differentiation and axon growth assay

The NPS loaded hydrogels were used for NSPCs differentiation and axon growth assay *in vitro*. Differentiation was induced by culturing neurospheres for 7 or 14 days in growth factor free medium. Axon growth was assessed by GAP43 immunostaining after 7 days of culture in proliferation medium. The differentiation medium was identical to the proliferation medium except that the growth factors were omitted. It consisted of DMEM/F12 supplemented with 1% non-essential amino acids, 1% sodium pyruvate, 1% penicillin-streptomycin, 1% GlutaMAX (Gibco, USA), 2% B27, and 6 g/L glucose. To enhance neuronal differentiation, 50 μM DAPT (Selleck, USA) was added (Xu et al., 2022). Three independent replicates were performed for each condition.

### 2.5 RT-qPCR

Total RNA was obtained from differentiated cells using TRIzol (Invitrogen, USA). Three independent biological replicates were analyzed, each with three technical replicates. First-strand cDNA was synthesized using a Hifair^®^ II 1st Strand cDNA Synthesis SuperMix for qPCR kit (YEASEN, 11123ES60). Quantitative PCR was performed using Hifair^®^ qPCR SYBR Green Master Mix (YEASEN, 11201ES08), and relative mRNA expression levels were determined using below primers.

GAPDH sense 5′ CATCACTGCCACCCAGAAGACTG 3′, GAPDH antisense 5′ ATGCCAGTGAGCTTCCCGTTCAG 3′;
TUJ1 sense 5′ GTCTCTAGCCGCGTGAAGTC 3′, TUJ1 antisense 5′ GCAGGTCTGAGTCCCCTACA 3′;
MAP2 sense 5′ TTCCACCGAGCAGTCTTTCT 3′, MAP2 antisense 5′ CAGCGCCTTCAAGCTTATTT 3′;
DAB1 sense 5′ CAGAGAGCCGGCACTATCTC 3′, DAB1 antisense 5′ CCCTCTCGACACATCATCCT 3′;
PPFIA2 sense 5′ AGTGGACACTCAACGCCAAAGC 3′, PPFIA2 antisense 5′ GCTTTATCCTCCCGTCCATCTTC 3′;
KCNJ10 sense 5′ AGCTGGTGCTGATCCTGAGT 3′, KCNJ10 antisense 5′ CCTCCAACTTGAGCTTCTCG 3′;
LRRK2 sense 5′ ATGCTGGTTTTGGGGTACAA 3′, LRRK2 antisense 5′ CATCGAGACTGCCTGATGTC 3′;
DHH sense 5′ CAATTTGCCCTTCTTCCTCA 3′, DHH antisense 5′ GACTCCAAGTGCTCCTCCTG 3′;
POU3F2 sense 5′ TGTTTGCCATTTCCTGAACA 3′, POU3F2 antisense 5′ CTGTGGCAGAGGCTAAGGTC 3′;
GAD1 sense 5′ GAACCGCGCACTCTTCTATC 3′, GAD1 antisense 5′ CCGTCCCAGATTCACTGTTT 3′;
TRIM67 sense 5′ GTGAGGACCCCTCCTTTCTC 3′, POU3F2 antisense 5′ GTCAAGGCTCCGAAGACAAG 3′;
SOX10 sense 5′ GCAGGAAGGGTTAGGGTAGG 3′, POU3F2 antisense 5′ GCGGAGAAAGGATCAGAGTG 3′;
PLXNA4 sense 5′ AGATGCCGGCAATAAGTGAC 3′, PLXNA4 antisense 5′ AAGCCAGTTTCTGCTTTCCA 3′;
CAPN-1 sense 5′ GGGCCAGGATTATGAAACGC 3′, CAPN-1 antisense 5′ GGGGTTTGACATCAGTTCCG 3′;
Sema3A sense 5′ CAGCTCCAGTTACCACACCT 3′, Sema3A antisense 5′ TTCCAGCCCATTTGCATTCA 3′;
ALK sense 5′ GGGCAGTAAGACGTTTTCCG 3′, Sema3A antisense 5′ AAACATTCCGGGCAGAGGAG 3′;
ATP8A2 sense 5′ TGTGTGATCTGAGCGTCTGG 3′, Sema3A antisense 5′ CATTGAGGCTTTCCCAGGGT 3′;
PPFIA2 sense 5′ TGTCTTTCTCCTCCCGTTGC 3′, Sema3A antisense 5′ GCCACTGCTTTGGGAACCC 3′.

### 2.6 Immunocytochemistry

Cells were fixed with 4% paraformaldehyde (PFA) at 4 °C overnight, rinsed 3 times with PBS, and permeabilized with 0.2% Triton X-100 in 5% bovine serum albumin/PBS for 60 min. The cells were then incubated with primary antibodies diluted in blocking solution for 1 h at room temperature (RT). After washing with PBS, cells were incubated with donkey Alexa Fluor conjugated secondary antibodies (1:500, Invitrogen) for 30 min at RT. Nuclei were counterstained with Hoechst 33342, and images of the stained cells were acquired using an Imager.Z2 fluorescence microscope (Zeiss, USA). Three independent replicates were performed, and at least four random fields were examined per sample.

### 2.7 Surgical procedures

A T11 complete transection SCI model was established in female C57BL/6 mice (6–8 weeks old, weighing 18–22 g) using microscissors, as previously described with slight modifications (Xu et al., 2022). Anesthesia was induced by intraperitoneal injection of sodium pentobarbital (50 mg/kg). After complete transection at T11, the lesion gap was filled with either CEC/PEG-(CHO)_2_ scaffold alone or NPS loaded scaffold. Mice were randomly assigned to five groups (*n* = 15 per group): Control group (surgery only), Scaffold group (CEC/PEG-(CHO)_2_ scaffold only), NPS group (5 μg NPS delivered locally), Functional scaffold group (CEC/PEG-(CHO)_2_ scaffold loaded with 5 μg NPS), and NPSR-KO group (NPSR1 gene knockout mice implanted with NPS loaded functional scaffold). For local delivery, NPS was dissolved to a concentration of 5 μg/μL, and 1 μL of the solution was absorbed into the scaffold, giving a local dose of 5 μg per animal (250 μg/kg based on an average body weight of 20 g). Additionally, mice received daily intraperitoneal injections of NPS (0.2 μg/μL, 100 μL, equivalent to 1 mg/kg) for three consecutive days.

### 2.8 Histology and immunohistochemistry

Mice were anesthetized by intraperitoneal injection of sodium pentobarbital (50 mg/kg body weight) and transcardially perfused with 0.9% NaCl, followed by fixation with 4% paraformaldehyde (PFA). Spinal cords were immediately dissected, post fixed overnight at 4 °C, and cryoprotected by sequential immersion in 20% and 30% sucrose solutions at 4 °C. Horizontal spinal cord segments (1 cm in length) containing the injury site were cut into 10 μm thick sections on a cryostat. Sections were permeabilized and blocked in PBS containing 5% bovine serum albumin and 0.1% Triton X-100 for 1 h at RT. The sections were then incubated overnight at 4°C with primary antibodies.

The following primary antibodies were used: Nestin (rabbit, Proteintech, 1:400; NSPCs), SOX2 (rabbit, Proteintech, 1:400; NSPCs), PAX6 (rabbit, Proteintech, 1:400; NSPCs), TUJ1 (mouse, Proteintech, 1:400; immature neurons), MAP2 (mouse, Abcam, 1:400; mature neurons), GFAP (rat, Thermo Fisher, 1:400; astrocytes), GAP43 (rabbit, Proteintech, 1:400; axons), MMP-9 (rabbit, Abclonal, 1:400; secreted MMP-9), TNF-α (mouse, Proteintech, 1:400; pro-inflammatory cytokines), IL-10 (mouse, Proteintech, 1:400; anti-inflammatory cytokines), CD68 (rat, Abcam, 1:400; activated immune cells), CSPGs (mouse, Cell Signaling Technology, 1:400; glial scar), synaptophysin (SYP, rabbit, Proteintech, 1:400; presynaptic membranes), and cAMP (rabbit, Proteintech, 1:400; intracellular second messenger).

After primary antibody incubation, sections were washed three times with PBS and then incubated for 1 h at RT with the following Alexa Fluor conjugated secondary antibodies (A21202, 488 donkey anti-mouse; A11077, 568 goat anti-rat; A10042, 568 donkey anti-rabbit; A11004, 568 goat anti-mouse) at 1:500 in PBS. Sections were washed three more times, mounted on glass slides, and counterstained with DAPI. Imaging was performed using an Observer.Z1 confocal microscope (Zeiss, USA) or an SP8 confocal microscope (Leica, USA).

### 2.9 Behavioral assessments

Locomotor recovery was assessed weekly using the Basso Mouse Scale (BMS) by two blinded observers. Each mouse was allowed to freely explore an open field, and hindlimb function was scored independently by both evaluators. Three mice per group were analyzed.

### 2.10 Electrophysiological test

Cortical motor evoked potentials (CMEPs) were assessed in mice 6 weeks after SCI with a Keypoint 9033A07 instrument (Alpine BioMed ApS, Denmark). Mice were anesthetized with sodium pentobarbital (50 mg/kg, i.p.) and placed in a prone position. The stimulating electrodes were placed subcutaneously: one behind the midline brow intersection and the other on the opposite side. A recording electrode was inserted into a hindlimb muscle (e.g., gastrocnemius), and a ground electrode was placed subcutaneously near the L1 spine. Stimulation parameters were set at 6.0 mA and 0.2 ms pulse duration. Each measurement was repeated five times to ensure waveform consistency. All mice in each group were tested.

### 2.11 Calcium ion probe staining

Calcium ion staining was performed using the Fluo-4 calcium detection kit (Biyuntian Biotechnology, China). Frozen sections were rewarmed to RT, washed with PBS or distilled water, and incubated with Fluo-4 staining solution at 37 °C for 30 min. Sections were then rinsed with PBS, counterstained with DAPI, washed again, and mounted. Green fluorescence indicating calcium ion expression was visualized with a fluorescence microscope (Zeiss, USA). Three mice per group were analyzed.

### 2.12 Statistical analysis

All experiments were conducted with at least three independent replicates (*n* ≥ 3). Data are presented as mean ± standard deviation (SD). Outliers, defined as data points falling outside the range of mean ± 2SD (*n* > 10) or mean ± 3SD (*n* ≤ 10), were excluded from the analysis. For comparisons between two groups, a two-tailed Student’s *t*-test was used. For comparisons across more than two groups, one-way (repeated-measures) ANOVA was applied, followed by Tukey’s *post hoc* test for detailed pairwise comparisons. Statistical significance was set at *P* < 0.05. *P < 0.05, **P < 0.01, ***P < 0.001.

## 3 Results and discussion

### 3.1 Fabrication and characterization of the NPS-loaded CEC/PEG-(CHO)_2_ hydrogel scaffold

Chitosan was modified with acrylic acid via Michael addition to yield CEC, which was then crosslinked with PEG-(CHO)_2_ through dynamic Schiff base linkages between the amino groups of CEC and the aldehyde groups of PEG-(CHO)_2_. The CEC/PEG-(CHO)_2_ hydrogels were prepared by mixing aqueous solutions of the two components at 37 °C. Notably, water was the only solvent used throughout the synthesis, so no organic solvent related toxicity was introduced. The resulting hydrogel was then loaded with NPS to obtain the functional scaffold (Figure 1a). The chemical structures of PEG-(CHO)_2_ and CEC are shown in Figure 1b, highlighting the functional groups involved in crosslinking. The successful chemical modification and network formation were further confirmed by FTIR spectroscopy (Figure 1c). The CEC spectrum showed a peak at 1573 cm^−1^, corresponding to the asymmetric stretching of COO^–^. The PEG-(CHO)_2_ spectrum had a distinct aldehyde *C* = O stretching band at 1725 cm^−1^. The formation of a Schiff base linkage between CEC and PEG-(CHO)_2_ was evidenced by a characteristic absorption peak at 1644 cm^−1^ in the hydrogel spectrum (Wei et al., 2015). Uniaxial compression tests revealed that the hydrogel is porous and soft (Figure 1d). Up to about 80%, the stress-strain curve exhibited an initial elastic region followed by a densification plateau. The maximum compressive stress was 0.124 MPa, and the elastic modulus was 0.081 MPa. The scaffold degraded gradually over 28 days (Figure 1e), and showed swelling kinetics suitable for spinal cord implantation (Figure 1f). The swelling ratio increased rapidly in the first few hours and then slowly approached equilibrium. SEM imaging revealed a porous microstructure (50−150 μm) enabling NPS diffusion (Figure 1g), and release profiling showed rapid NPS delivery: about 80% was released within 10 h, and release was completed by 24 h (Figure 1h). The CEC/PEG-(CHO)_2_ hydrogel exhibited excellent injectability and fluidity prior to gelation, enabling effective filling of irregularly shaped injury sites (Figures 1k, l) and close apposition to the surrounding tissue, thereby substantially minimizing additional trauma to the adjacent vulnerable neural tissue. Upon crosslinking, its self-healing capability was confirmed by the tight union of distinctively stained gel pieces, which remained intact against gravitational forces (Figures 1i, j). This reversible recovery feature allows the hydrogel to adjust to the dynamic spinal microenvironment, automatically mend stress-induced microcracks, and thereby sustain its structural integrity and load-bearing function. Figure 1m illustrates the internal crosslinking network. We also performed AO/PI staining and CCK-8 assays to assess biological safety of the CEC/PEG-(CHO)_2_ hydrogel or NPS (Supplementary Figure 1). Primary neurospheres cultured with the scaffold, NPS (100 ng/mL), or scaffold loaded with NPS (100 ng/mL) remained viable after 7 days, indicating that neither the scaffold nor NPS exhibited significant cytotoxicity toward NSPCs. All three groups also exhibited good cell viability in the CCK-8 experiment.

**FIGURE 1:**
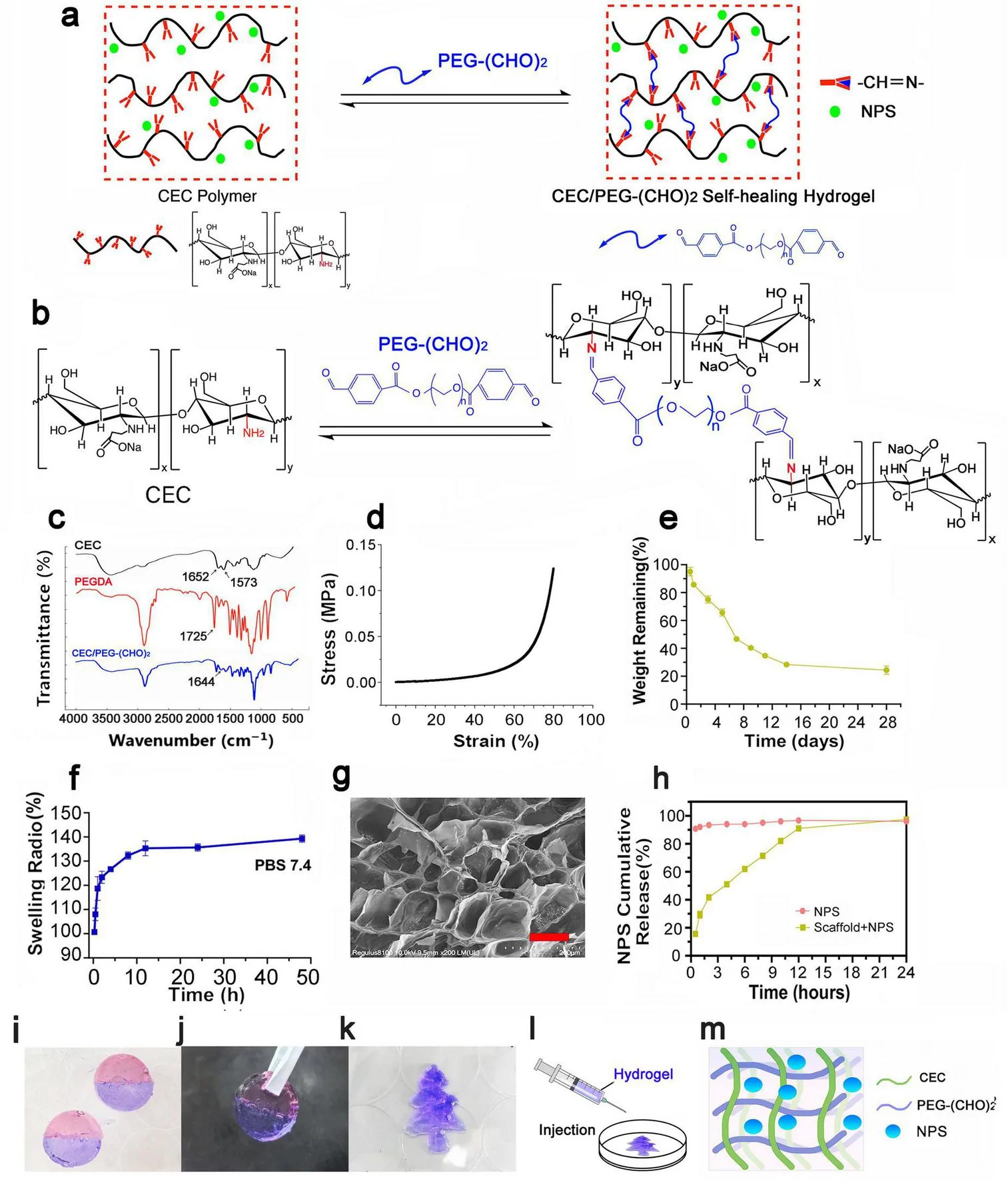
Fabrication and characterization of the NPS loaded CEC/PEG-(CHO)_2_ hydrogel scaffold. **a)** The CEC/PEG-(CHO)_2_ hydrogel was formed via Schiff-base reaction between the amine groups of CEC and the aldehyde groups of PEG-(CHO)_2_, followed by loading of NPS into the scaffold. **(b)** Chemical structures of PEG-(CHO)_2_ and CEC, with the functional groups involved in crosslinking highlighted. **(c)** FTIR Spectra of the CEC Polymer, PEG-(CHO)_2_ Polymer, and the CEC/ PEG-(CHO)_2_ Hydrogel. **(d)** Uniaxial compression tests revealed characteristic mechanical behavior of the CEC/PEG-(CHO)_2_ hydrogel. **(e)** Degradation profile of the CEC/PEG-(CHO)_2_ scaffold over 28 days. **(f)** Swelling ratio of the functional scaffold within 50 h. **(g)** SEM image showing porous microstructures of the CEC/PEG-(CHO)_2_ scaffold. Scale bar = 100 μm. **(h)** Release profile of NPS from the functional scaffold over 24 h. **(i)** Macroscopic images showing the self-healing capability of the CEC/PEG-(CHO)_2_ hydrogel. Hydrogels were cut into equal 2 pieces and put together, one stained with crystal violet and the other stained with Sudan red. **(j)** The hydrogels healed completely into one block after 2.5 h at 25°C. **(k)** The photograph of the self-healing hydrogel passing through a needle without clogging. **(l)** Scheme of the injection process of the CEC/PEG-(CHO)_2_ hydrogel. **(m)** Internal crosslinking network of CEC/PEG-(CHO)_2_ hydrogel.

The rapid release of NPS (Figure 1h) is primarily driven by diffusion through the porous hydrogel network rather than polymer degradation. As noted by Pogostin et al., small molecules diffuse quickly through the porous nanostructure of peptide hydrogels, limiting their ability to extend release (Pogostin et al., 2025). Indeed, NPS (about 2 kDa) is much smaller than the pore size of our scaffold (50–150 μm, Figure 1g), making diffusion the dominant mechanism. In contrast, the gradual mass loss in Figure 1e reflects hydrolytic cleavage of Schiff base crosslinks, a much slower process. Importantly, drug release and bulk degradation do not need to occur on the same timescale. As Malu et al. recently demonstrated, hydrogels can achieve controlled release through changes in mesh size and diffusivity well before complete degradation (Malu et al., 2025). The relatively rapid release of NPS from the scaffold is intentionally designed to match the acute inflammatory window after SCI, which peaks within 72 h (Hellenbrand et al., 2024; Kwon et al., 2011). Thus, fast NPS delivery targets this critical window, when anti-inflammatory and neuroprotective intervention is most needed, while sustained scaffold mass retention provides long-term structural support for nerve regeneration (Quan et al., 2026). These characteristics allow the scaffold to fill irregular lesion cavities, provide structural support, and deliver NPS locally to help modulate the hostile post SCI microenvironment.

### 3.2 Phenotypic characterization of mouse NSPCs and the pro-neurogenic effects of NPS *in vitro*

Primary NSPCs were isolated from embryonic day 15 mouse telencephalons. To confirm their characteristics *in vitro*, the isolated NSPCs were cultured in proliferation medium supplemented with epidermal growth factor and basic fibroblast growth factor. After 7 days, the cells formed floating neurospheres (Figure 2a). Immunostaining revealed strong expression of the NSPCs markers Nestin (Figure 2b), PAX6 (Figure 2c), and SOX2 (Figure 2d). Quantitative immunofluorescence demonstrated that 89.5% ± 3.5% of NSPCs expressed Nestin (Figure 2e), 91.1% ± 1.6% expressed PAX6 (Figure 2f), and 89.9% ± 2.7% expressed SOX2 (Figure 2g), indicating that the isolated cells were predominantly NSPCs. A schematic diagram of the isolation procedure is shown in Figure 2h. These results confirm that our isolation protocol yields a homogenous population of NSPCs, a prerequisite for subsequent differentiation and axon growth assays. The high expression of Nestin, SOX2, and PAX6 is consistent with previous reports on embryonic NSPCs identity, providing a reliable cellular model to investigate the effects of NPS on neuronal differentiation and regeneration.

**FIGURE 2:**
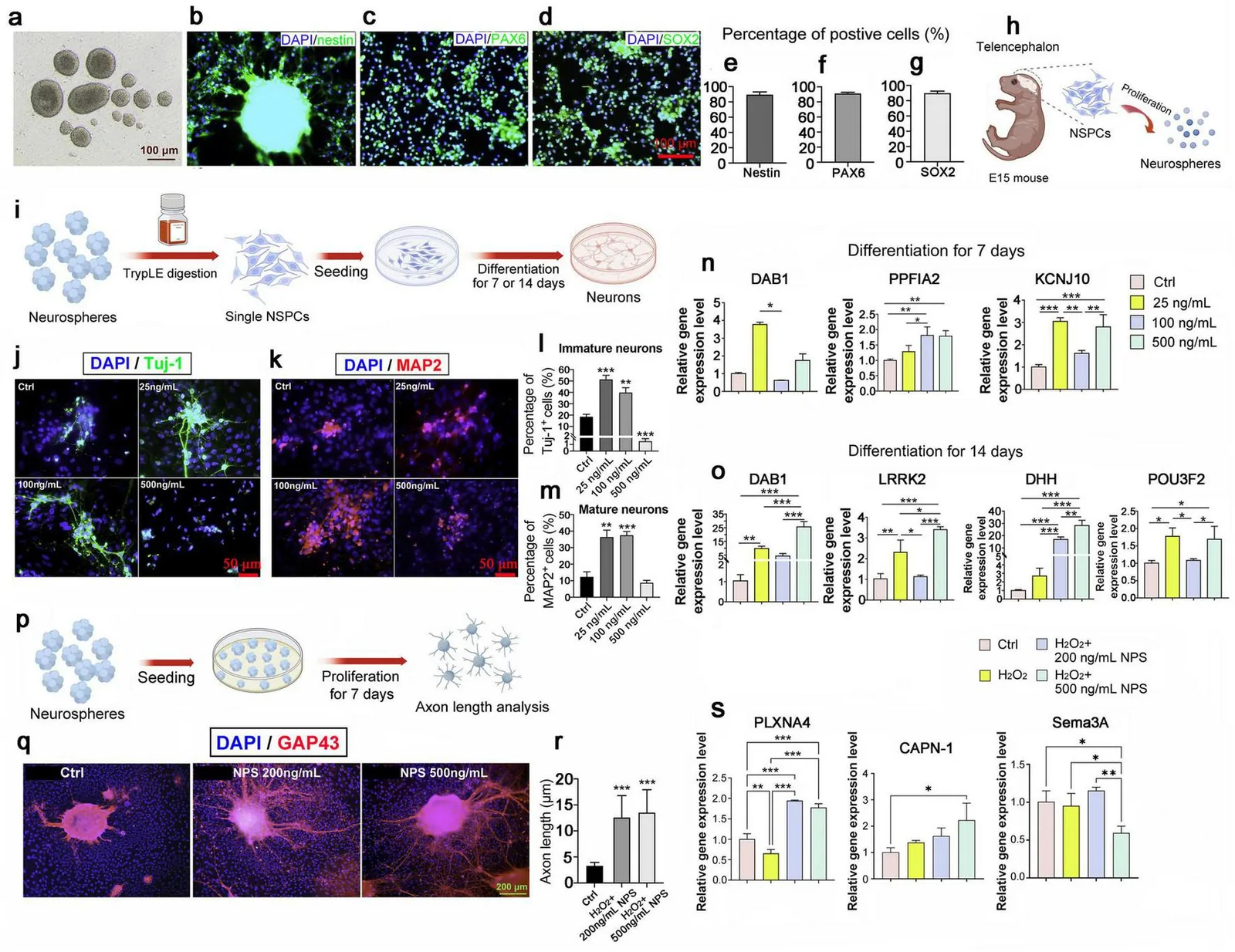
Phenotypic characterization of mouse NSPCs and the Pro-neurogenic effects of NPS loaded CEC/PEG-(CHO)_2_ hydrogel scaffold *In Vitro.* **(a)** After 7 days in proliferation medium, isolated NSPCs formed neurospheres. Scale bar = 100 μm. **(b–d)** Neurospheres were positive for the NSPC markers Nestin, SOX2, and PAX6. Scale bar = 100 μm. **(e–g)** Quantification of Nestin^+^, SOX2^+^, and PAX6^+^ cells. **(h)** Schematic diagram of NSPC isolation. **(i)** Schematic of the NSPC differentiation assay. **(j)** After 7 days of differentiation, immature neurons were immunostained for Tuj-1. Scale bar = 50 μm. **(k)** After 14 days, mature neurons were immunostained for MAP2. Scale bar = 50 μm. **(l,m)** Quantification of Tuj-1^+^ immature neurons **(l)** and MAP2^+^ mature neurons **(m)**. **P < 0.01, ***P < 0.001. **(n,o)** qPCR analysis of transcription factors associated with neuronal differentiation at 7 **(n)** and 14 **(o)** days. *P < 0.05, **P < 0.01, ***P < 0.001. **(p)** Schematic of the NSPC axon growth assay. **(q)** After 7 days in proliferation culture, axons were immunostained for GAP43. Scale bar = 200 μm. **(r)** Quantification of axon length. **(s)** qPCR analysis of transcription factors regulating axon growth. *P < 0.05, **P < 0.01, ***P < 0.001.

To assess whether NPS loaded scaffold influences neuronal differentiation and axon growth, NSPCs were dissociated with TrypLE and plated in dishes or 48-well plates (Figure 2i). Neuronal differentiation was quantified by staining for the immature neuronal marker TUJ1 (βIII-tubulin) and the mature neuronal marker MAP2. After 7 days of differentiation, the proportion of TUJ1^+^ neurons was significantly higher in NPS-treated cultures. Specifically, 25 ng/mL and 100 ng/mL NPS increased TUJ1^+^ neurons to 51.3% ± 3.7% (*P* < 0.001) and 39.8% ± 4.3% (*P* < 0.01), respectively, compared with 18.6% ± 2.1% in controls (Figures 2j, l). This dose dependent enhancement suggests that NPS, within a proper concentration range, robustly stimulates early neuronal commitment. By contrast, 500 ng/mL NPS strongly suppressed neuronal differentiation, reducing TUJ1^+^ neurons to 1.5% ± 0.4% (*P* < 0.001; Figures 2j, l), indicating a toxic effect at supra doses. After 14 days of differentiation, the proportion of MAP2^+^ mature neurons was also elevated in the 25 ng/mL and 100 ng/mL NPS groups, reaching 36.4% ± 4.2% (*P* < 0.01) and 37.5% ± 2.3% (*P* < 0.001), respectively, compared with 12.3% ± 3.1% in controls (Figures 2k, m). These results confirm that NPS not only initiates neuronal differentiation but also supports neuronal maturation, exhibiting its potential as a pro-neurogenic factor.

To further examine NPS-mediated effects, we analyzed the expression of transcription factors associated with neuronal fate by qPCR. At day 7, 25 ng/mL NPS significantly increased DAB1 expression compared with controls (Figure 2n), consistent with its role in suppressing astrocytic differentiation (Xu et al., 2022). Both 25 ng/mL and 100 ng/mL NPS upregulated PPFIA2, which encodes liprin-α-2, a key regulator of excitatory synapse density and vesicle dynamics (Spangler et al., 2013), as well as KCNJ10, a critical channel mediating oligodendrocytic K^+^ conductance and essential for myelination (Neusch et al., 2001; Figure 2n). These changes suggest that NPS may also promote synaptic integration and myelination. After 14 days, the same NPS doses upregulated DAB1, LRRK2, DHH, and POU3F2 (Figure 2o). Notably, LRRK2 regulates synapse morphology (Lee et al., 2012), DHH encodes a Schwann cell-derived protein essential for perineurium formation (Jung et al., 2015), and POU3F2, a class III POU transcription factor, is critical for Schwann cell myelination (Ghislain and Charnay, 2006). Collectively, these transcriptional profiles suggest that NPS facilitates the coordinated development of functional neurons, which is of particular interest for neural network repair.

The effect of NPS on axon growth was assessed by GAP43 immunostaining, a marker of axonal growth and regeneration (Figure 2p). After 7 days of proliferation, only a few GAP43^+^ axons extended from neurospheres in controls (Figure 2q). However, in the 200 ng/mL and 500 ng/mL NPS groups, axon length increased markedly to 12.5 ± 4.4 μm (*P* < 0.001) and 13.3 ± 4.5 μm (*P* < 0.001), compared with 3.2 ± 0.8 μm in controls (Figure 2r). Of note, even at higher concentrations that suppressed neuronal differentiation, NPS still potently stimulated axonal outgrowth. qPCR further confirmed that NPS counteracted H_2_O_2_-induced inhibition of axonal growth, accompanied by upregulation of axon-associated genes including PLXNA4, CAPN-1, and Sema3A (Figure 2s). These genes are involved in axon guidance, cytoskeletal remodeling, and semaphorin signaling, indicating that NPS may enhance regenerative capacity by modulating axonal growth pathways. Together, these *in vitro* findings establish NPS as a dual-function modulator that enhances both neuronal differentiation and axonal outgrowth of NSPCs, providing an experimental foundation for subsequent *in vivo* assays in SCI repair.

### 3.3 Microenvironment-modulating function of the functional CEC/PEG-(CHO)_2_ hydrogel scaffold after SCI

To evaluate the therapeutic efficacy of NPS *in vivo*, we employed a T11 complete transection model in C57BL/6 mice, using a pH-responsive CEC/PEG-(CHO)_2_ hydrogel scaffold as the NPS carrier. Our previous studies indicate that CEC/PEG-(CHO)_2_ scaffolds mitigate oxidative stress and facilitate nerve regeneration by adapting their physicochemical properties to the local microenvironment to release bioactive molecules (Gao et al., 2025). A schematic of the transplantation procedure was provided in Figure 3a. MMP-2 and MMP-9, abundantly expressed by activated astrocytes, contribute to secondary injury through inflammatory signaling and extracellular matrix degradation (Asano et al., 2022; Hsu et al., 2008). At 3 days post injury, the NPS functionalized scaffold induced the most pronounced reduction in MMP-9^+^ area (16.4% ± 4.5%), significantly greater than reductions achieved by the scaffold alone (27.5% ± 2.8%) or free NPS (34.4% ± 2.5%), compared with the control group (42.6% ± 7.1%) (Figures 3b, c). Notably, when the same scaffold was transplanted into NPSR-KO mice, no reduction in MMP-9 deposition was observed, indicating that this effect depends on NPSR signaling. The genotyping data for the NPSR-KO gene knockout mice was presented in Supplementary Figure 2.

**FIGURE 3:**
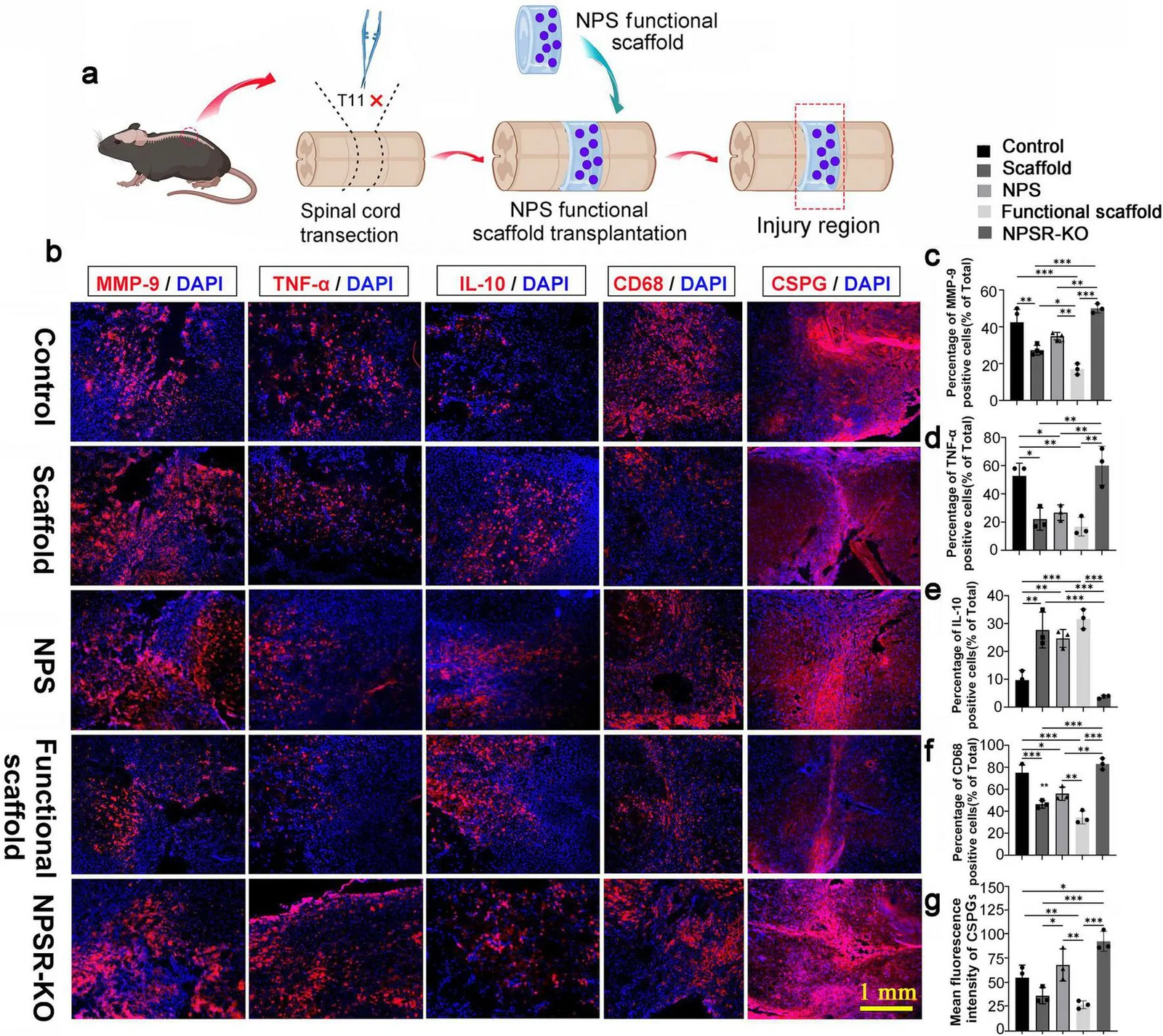
The Functional scaffold mitigated the inflammatory microenvironment, reduced matrix metalloproteinase accumulation, and attenuated glial scar formation in SCI mice. **(a)** Schematic of SCI transection model and scaffold transplantation. **(b)** At 3 days post surgery, tissues were immunostained for MMP-9 (indicating MMP accumulation), TNF-α (pro-inflammatory), IL-10 (anti-inflammatory), and CD68 (marking microglia/macrophages). At 6 weeks post injury, tissues were immunostained for CSPGs (indicating glial scars). Scale bar = 1 mm. **(c–g)** Quantification of MMP-9^+^ cells, TNF-α^+^ cells, IL-10^+^ cells, CD68^+^ cells, and CSPGs^+^ area. *P < 0.05, **P < 0.01, ***P < 0.001. Groups (left to right): control, scaffold, NPS, functional scaffold and NPSR-KO.

Activated immune cells release pro-inflammatory cytokines (peaking at 3 days post injury) and exacerbate tissue damage (Sterner and Sterner, 2023). We therefore examined TNF-α, IL-10, and CD68 expression at 3 days post injury (Figures 3b, d–f). The functional scaffold group exerted the best outcome: it markedly reduced TNF-α^+^ cells (16.6% ± 6.6%, *P* < 0.01) or CD68^+^ activated microglia/macrophages (33.8% ± 5.4%, *P* < 0.001) compared with the control group (53.6% ± 9.5%, 75.3% ± 7.6%, respectively). Concurrently, it promoted the highest expression of the anti-inflammatory cytokine IL-10 (31.8% ± 3.8% vs. 9.4% ± 3.6% in controls, *P* < 0.001). By contrast, the NPSR-KO group failed to reduce TNF-α^+^ or CD68^+^ microglia/macrophages and showed very low local expression of IL-10 (3.7% ± 0.5%).

Because persistent inflammation and activated microglia/macrophages contribute to glial scar formation, we assessed chondroitin sulfate proteoglycans (CSPGs) at 6 weeks post injury (Kisucká et al., 2026; Zheng et al., 2026). The functional scaffold group significantly reduced CSPGs deposition (mean fluorescence intensity 27.8 ± 4.2 vs. 47.7 ± 9.8 in controls, *P* < 0.01) (Figures 3b, g). However, this scar-reducing effect was abolished in NPSR-KO mice, confirming that the benefits are specifically mediated through the NPS-NPSR axis.

Collectively, these results indicate that the NPS functionalized scaffold remodels the post SCI microenvironment by attenuating inflammation, reducing MMPs accumulation, and limiting glial scar formation, changes that are known to support neurogenesis and axon regeneration. Thus, this strategy may help overcome a major challenge in SCI repair: the tendency of endogenous NSPCs to differentiate into glial rather than neuronal lineages under hostile conditions (Anjum et al., 2020).

### 3.4 The functional CEC/PEG-(CHO)_2_ hydrogel scaffold enhances neurogenesis and myelination by recruiting endogenous NSPCs

To test whether the functional scaffold recruits endogenous NSPCs and promotes their neuronal differentiation, we first examined the lesion site at 7 days post injury. The functional scaffold significantly enhanced the recruitment of NSPCs, yielding a higher proportion of Nestin^+^ cells (21.2% ± 3.2%) compared with the scaffold alone (13.7% ± 2.0%) or free NPS (8.4% ± 2.9%) (Figures 4a, e). This effect likely results from the scaffold’s functions: its porous microstructure supports cell infiltration and retention, while the PEG-(CHO)_2_ component contains RGD like sequences that serve as cell-binding motifs. These RGD motifs are known to promote cell adhesion and migration by interacting with integrins (e. g., αvβ3 and α5β1) expressed on NSPCs (Hao et al., 2024; Pally et al., 2025), potentially amplifying the neurogenic potential of NPS. Tuj-1 staining further confirmed the neuronal presence at 7 days, with the functional scaffold group showing the highest proportion of Tuj-1^+^ neurons (29.0% ± 2.7%), compared with the scaffold (16.9% ± 2.1%) or NPS (22.3% ± 5.5%) groups (Figures 4b, f). Tuj-1 expression remained very low in NPSR-KO mice (1.9% ± 0.1%), underscoring that this pro-neurogenic effect depends specifically on NPSR signaling.

**FIGURE 4:**
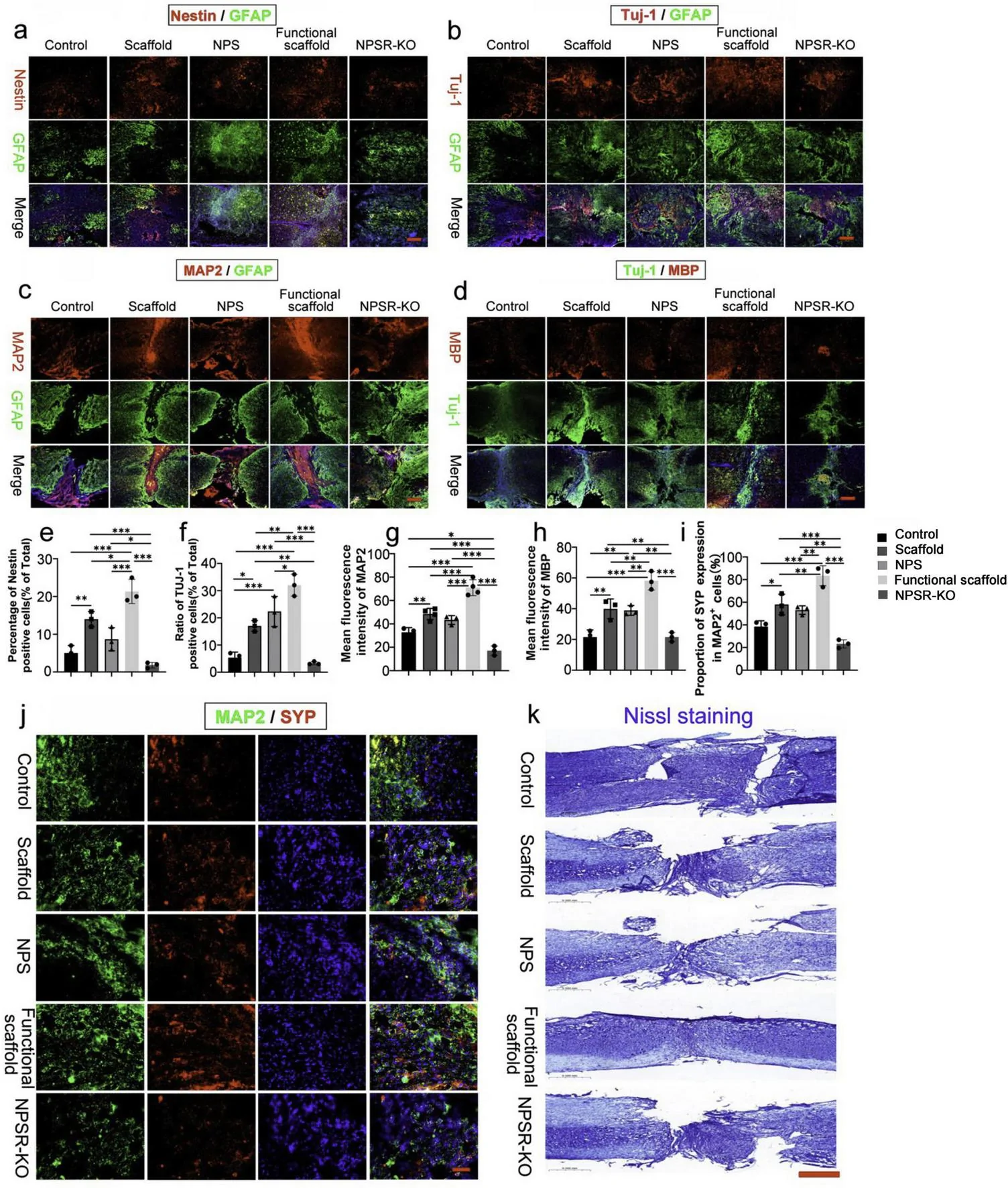
The functional scaffold enhanced neuronal differentiation and myelination of endogenous NSPCs after SCI. **(a,b)** At 7 days post injury, the functional scaffold recruited endogenous Nestin^+^ NSPCs to the lesion site and promoted their differentiation into newly generated Tuj-1^+^ neurons. Scale bar = 500 μm. **(c,d)** At 6 weeks post injury, the functional scaffold promoted neural regeneration and myelination in the lesion area, with increased MAP2^+^ mature neurons and MBP^+^/Tuj-1^+^ co-localization. Scale bar = 500 μm. **(e,f)** Quantification of Nestin^+^ and Tuj-1^+^ cells. *P < 0.05, **P < 0.01, ***P < 0.001. Groups (left to right): control, scaffold, NPS, functional scaffold and NPSR-KO. **(g,h)** Quantification of MAP2 and MBP fluorescence intensity. *P < 0.05, **P < 0.01, ***P < 0.001. Groups (left to right): control, scaffold, NPS, functional scaffold and NPSR-KO. **(i)** Quantification of SYP expression in MAP2^+^ cells. *P < 0.05, **P < 0.01, ***P < 0.001. Groups (left to right): control, scaffold, NPS, functional scaffold and NPSR-KO. **(j)** At 6 weeks, SYP/MAP2 double-positive cells were evident at the lesion site in the functional scaffold group. Scale bar = 50 μm. **(k)** Nissl staining revealed no residual scaffold material at the lesion site at 6 weeks post implantation, indicating complete degradation. Scale bar = 500 μm.

Long term regeneration assessed at 6 weeks post SCI revealed that the functional scaffold group sustained superior neuronal regeneration, exhibiting the highest density of MAP2^+^ mature neurons (71.5% ± 7.1%) compared with all other groups (control: 32.3% ± 4.7%; scaffold: 48.2% ± 5.4%; NPS: 42.9% ± 4.4%) (Figures 4c, g). The near absence of MAP2^+^ neurons in NPSR-KO mice further confirms the essential role of the NPS-NPSR axis in sustaining neuronal regeneration.

Intact neuronal axons are ensheathed by myelin to accelerate signal transmission. We therefore assessed axonal ensheathment in newly generated neurons. Myelination was significantly enhanced in the functional scaffold group, as shown by higher MBP fluorescence intensity (57.7 ± 6.6) than in scaffold (39.5 ± 6.9) or NPS (38.2 ± 3.4) groups (Figures 4d, h). Increased myelination suggests that neurons induced by the NPS functionalized scaffold not only survive but also attract oligodendrocyte support. In contrast, the NPSR-KO group showed weak mean fluorescence intensity of MBP in the damage zone.

To determine whether these MAP2^+^ neurons matured into functional units, we examined presynaptic formation. The functional scaffold group showed a significantly higher proportion of MAP2^+^/SYP^+^ neurons (83.0% ± 8.9%) compared with control group (38.8% ± 5.0%, *P* < 0.001), scaffold group (57.1% ± 9.0%, *P* < 0.01) or NPS group (52.4% ± 4.1%, *P* < 0.01) (Figures 4i, j). The high degree of synaptic colocalization indicates that mature neurons are integrating into local circuits. Patch-clamp recordings at 6 weeks post injury showed that neurons in the functional scaffold group had significantly higher action potential frequency than scaffold and NPS groups, matching control levels. This indicates that the functional scaffold supports enhanced neuronal excitability (Supplementary Figure 3).

Nissl staining at 6 weeks showed no residual scaffold material at the lesion site, indicating complete scaffold degradation. Furthermore, increased cellular infiltration and reduced cavity formation were observed in the lesion core (Figure 4k). In the NPSR-KO group, SYP^+^/MAP2^+^ cells were scarce, and Nissl staining revealed poor integration between the lesion area and the surrounding spinal cord tissue. Collectively, these results demonstrate that the NPS functionalized scaffold achieves a vital therapeutic goal: it recruits endogenous NSPCs to the injury site, directs their sustained differentiation into mature neurons, promotes myelination, and supports synaptic integration. This progression, from recruitment to circuit integration, does not merely replace lost cells, but reconstructs functional neural networks within a favorable microenvironment.

### 3.5 The functional CEC/PEG-(CHO)_2_ hydrogel scaffold improves motor recovery via NPSR-mediated Ca^2 +^/cAMP signaling

To evaluate motor function recovery, we performed BMS open-field scoring over 6 weeks (Figure 5a).

**FIGURE 5:**
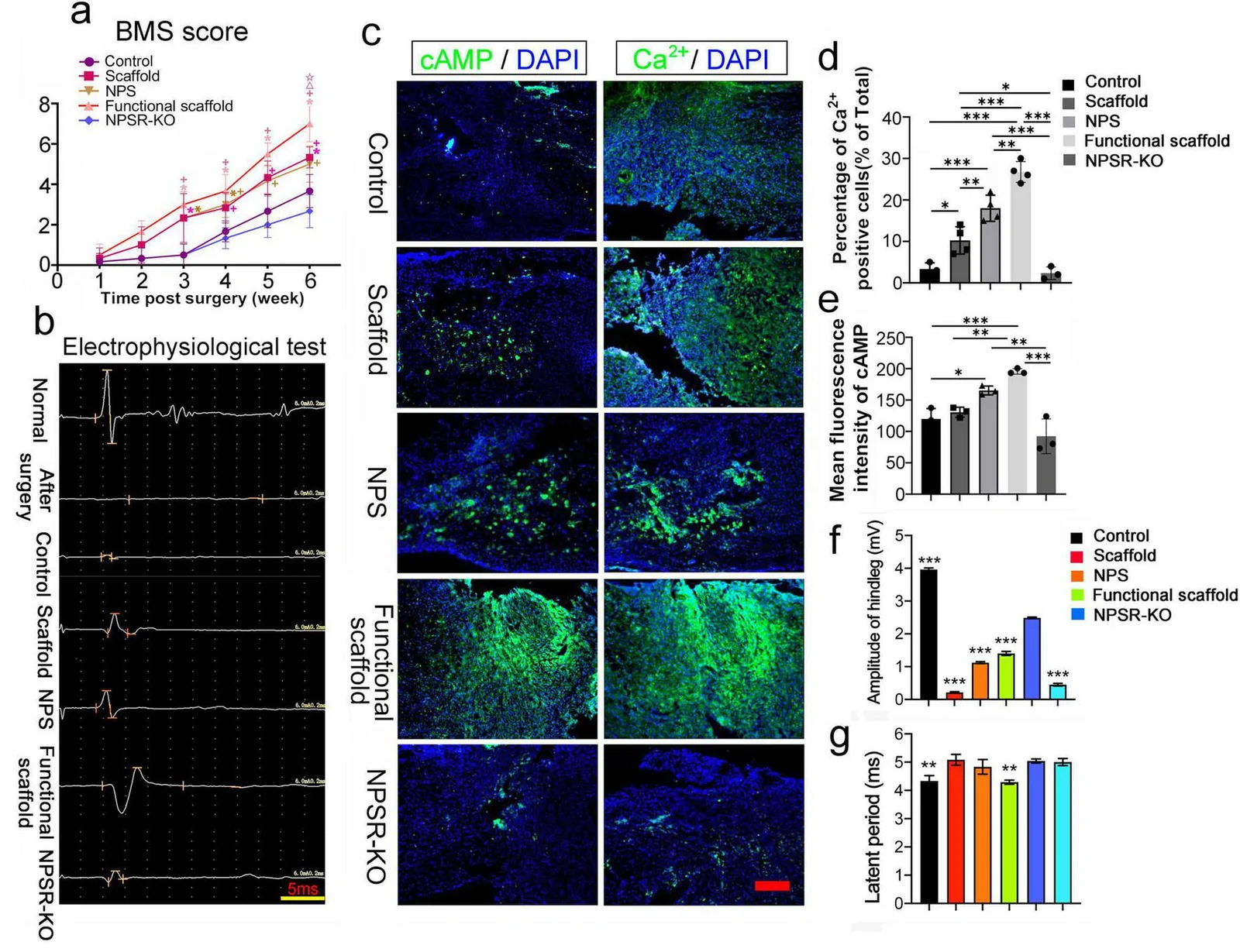
Functional scaffold transplantation improved hindlimb motor recovery, electrophysiological transduction and activated Ca^2 +^-cAMP signaling in SCI mice. **(a)** BMS open-field scores of mice receiving different treatments over 6 weeks. * vs. control group; + vs. NPSR-KO group; △ vs. NPS group; □ vs. scaffold group. **(b)** Electrophysiological test of the following groups: normal, immediately after surgery, control, scaffold, NPS, functional scaffold and NPSR-KO. Scale bar = 5 ms. Stimulus parameter: 6.0 mA, 0.2 ms. **(c)** Calcium ion probe staining for Ca^2 +^ and immunofluorescence for cAMP showed significant activation in the functional scaffold group. Scale bar = 500 μm. **(d,e)** Quantification of Ca^2 +^ signals and mean fluorescence intensity of cAMP. *P < 0.05, **P < 0.01, ***P < 0.001. Groups from left to right are: control, scaffold, NPS, functional scaffold and NPSR-KO. **(f,g)** Quantification of amplitude of hindleg and signal-to-response latency of each group. **P < 0.01, ***P < 0.001, both compared with functional scaffold group. Groups from left to right are: normal, control, scaffold, NPS, functional scaffold and NPSR-KO.

From 3 to 5 weeks post injury, the functional scaffold group showed significantly higher BMS scores than the control group and the NPSR-KO group. At 6 weeks post injury, mice implanted with NPS-functionalized scaffolds achieved a mean BMS score of 6.93 ± 0.76, significantly higher than that of the other four groups, indicating frequent or consistent plantar stepping with mostly coordinated paw placement and proper rotation. In contrast, the control, scaffold, or free NPS groups scored significantly lower (3.62 ± 0.66, 5.28 ± 0.34, and 4.98 ± 0.88, all *P* < 0.05). This superior recovery likely reflects restoration of local neural circuits. The lack of similar improvement in NPSR-KO mice confirms that these benefits are specifically mediated by the NPS-NPSR axis.

Electrophysiological assessment corroborated these functional improvements. Cortical motor evoked potentials (CMEPs) were recordable in all groups (Figure 5b). The functional scaffold group exhibited the largest response amplitude (2.49 ± 0.02 mV), significantly greater than the scaffold-only (1.12 ± 0.02 mV) and free NPS (1.40 ± 0.05 mV) groups (*P* < 0.001; Figure 5f). Amplitudes in the NPSR-KO group (0.45 ± 0.03 mV) and control group (0.21 ± 0.02 mV) were markedly reduced. No significant differences in response latency were observed among groups (Figure 5g). These electrophysiological data confirm that the functional scaffold restores motor function by reestablishing descending motor pathways.

To investigate the underlying signaling mechanisms, we firstly performed immunofluorescence staining to verify NPSR expression in NSPCs and injured spinal cord tissue. Specifically, we stained NSPCs in proliferating neurospheres *in vitro*, and examined spinal cord sections from control and NPS-treated mice at 3 days post injury. Our results show that neurospheres indeed express NPSR, with staining detected in both the cell membrane and cytoplasm (Supplementary Figure 4). Regarding tissue staining, NPSR expression was nearly undetectable in the normal mice spinal cord. However, at 3 days after SCI, NPSR-positive cells appeared in the lesion area of control group, which we interpret as activated endogenous NSPCs proliferating and migrating into the injured site (Supplementary Figure 5). Furthermore, NPS-treated mice exhibited stronger NPSR expression in the lesioned spinal cord. Together, these findings support that the therapeutic effects are mediated via the NPS-NPSR pathway.

We examined Ca^2 +^ and cAMP pathways, both involved in neuronal plasticity and repair. Intracellular Ca^2 +^ levels (Fluo-4 AM fluorescence) were significantly elevated in the functional scaffold group (26.4% ± 2.6%) compared with the control (3.5% ± 1.6%), scaffold-only (10.1% ± 3.4%), and free NPS (17.9% ± 2.9%) groups (Figures 5c, d). Similarly, cAMP immunostaining intensity was highest in the functional scaffold group (193.9 ± 5.1), significantly exceeding all other groups (Figures 5c, e). This dual activation is consistent with NPSR signaling through both Gq and Gs proteins (Shirsath et al., 2024). Extracellular signals activate convergent Ca^2 +^-CaMKIV and cAMP-PKA pathways that phosphorylate CREB. Phosphorylated CREB recruits CBP/p300 to upregulate regeneration-associated genes (e.g., Arginase I, *IL-6*), counteracting myelin inhibition. PKA also modulates Rho GTPases to facilitate cytoskeletal reorganization and axon regeneration (Guo et al., 2021; Servillo et al., 2002). A comprehensive mechanistic diagram of the NPS-NPSR-Ca^2 +^/cAMP signaling pathway is presented in Supplementary Figure 6. In addition, to assess *in vivo* systemic toxicity assessment, we performed H&E staining on major organs, including the heart, liver, spleen and kidneys, from mice in each treatment group at 6 weeks after SCI. As shown in Supplementary Figure 7, none of the treated SCI mice exhibited any obvious pathological changes in these organs under the conditions tested. This confirms the favorable systemic biocompatibility of the hydrogel/NPS system.

Collectively, these results establish the NPS functionalized hydrogel as a multifunctional platform for SCI repair. By remodeling the inhibitory microenvironment and activating NPS-NPSR signaling, it coordinates cellular repair processes, leading to improved motor and electrophysiological outcomes. This demonstrates the translational promise for severe SCI.

## 4 Conclusion

Implantation of NPS loaded CEC/PEG-(CHO)_2_ hydrogel scaffolds into mice with complete spinal cord transection at T11 effectively mitigated the inhibitory microenvironment. By activating the NPS receptor NPSR and its downstream Ca^2 +^ and cAMP signaling pathways, the functional scaffold recruited endogenous NSPCs to the lesion area, promoted their differentiation into functional neurons, and supported axonal regeneration. Consequently, this led to the partial reestablishment of neural circuitry and significant improvement in hindlimb motor function. Together, these results indicate that NPS functionalized scaffolds represent a promising translational therapeutic strategy for SCI repair.

## Data Availability

The original contributions presented in this study are included in this article/Supplementary material, further inquiries can be directed to the corresponding authors.
