# Supplementary material for: A pH-responsive and self-healing CEC/PEG-(CHO)2 hydrogel for controlled neuropeptide S delivery and endogenous neural repair after spinal cord injury
Source: Front Neurosci. 2026 Sep 16;20:1933351. doi: 10.3389/fnins.2026.1933351 (PMC13624114; doi:10.3389/fnins.2026.1933351)
Supplement: Supplementary file 1 [file Data_Sheet_1.docx]

Supplementary figure 1. Confocal Image of AO/PI Live/dead Staining of Neurospheres.

a-c AO/PI Live/dead staining of neurospheres (AO, green, live cells; PI, red, dead cells) cultured with scaffold, NPS (100ng/mL) or CEC/PEG-(CHO)_2_ scaffold loaded with NPS (100ng/mL) for 7 days. Scale bar = 50 μm.

d CCK-8 assay evaluating the cytocompatibility of hydrogel, NPS, and NPS loaded hydrogel on NSPCs.

Supplementary figure 2. Genotyping Analysis of NPSR‑KO Mice.

1-5 represents 5 mice samples. Homozygotes show only the 476 bp band; heterozygotes show three bands (476 bp, 858 bp, and 2080 bp); wildtype mice show two bands (858 bp and 2080 bp). all five mice were genotyped as homozygotes.

Supplementary figure 3. Functional Patch Clamp Recordings of Neuronal Excitability in Spinal Cord Slices at 6 weeks Post Injury

a Pictures of spinal cord neurons from 4 groups during recording.

b Example traces from current-clamped neurons from 4 groups.

c Comparison of the frequency of action potentials (APs) in 4 groups. control vs scaffold: *p = 0.0416, control vs NPS: *p = 0.0147, control vs functional scaffold: p = 0.9976, scaffold vs NPS: p = 0.9459, scaffold vs functional scaffold: *p = 0.0256, NPS vs functional scaffold: **p = 0.0088, F(3, 60) = 6.073, n = 5 neurons in control group, n = 5 neurons in scaffold group, n = 4 neurons in NPS group, n = 5 neurons in functional scaffold group, two-way ANOVA. * *P* < 0.05*, **P* < 0.01, ns = not significant.

Supplementary figure 4. Confocal Image of NPSR Expression of a Neurosphere after Cultured for 7 Days *in vitro*.

Scale bar = 100 μm.

Supplementary figure 5. NPSR Expression in Cells Within the Injured Area at 3 Days Post Injury in the Normal Group, Control Group, and NPS Group

a Western blotting data showing increased NPSR protein level in Control group and NPS group.

b Statistical graph of protein levels in the Normal group, Control group, and NPS group. ***P* < 0.01, ****P* < 0.001.

c Immunofluorescence staining of NPSR in the lesion area in Normal group, Control group or NPS group. Scale bar = 1 mm.

Supplementary figure 6. A Schematic Diagram Summarizing the Proposed Mechanism of NPS-mediated Regeneration via the NPSR→Ca²⁺/cAMP Signaling Axis

a Molecular signaling pathway.

b *In vivo* repair process summarizing how the functional scaffold attenuates immune reactivity (decreased TNF-α and CD68), decreases MMPs deposition, recruits endogenous NSPCs to the damage zone, and promotes their differentiation into mature, myelinated and synaptically integrated neurons.

c Functional outcomes highlighting the resulting improvements in motor recovery (BMS score) and electrophysiological function.

Supplementary figure 7. HE Staining Results at 6 Weeks Post Injury in Control, Scaffold, NPS, Functional Scaffold, and NPSR-KO Groups

Scale bar = 200 μm (liver, heart and spleen) and 400 μm (kidney).
